# Common Environmental Hazards and Male Infertility: Effects on Epididymal Immune Microenvironment

**DOI:** 10.3390/toxics14020171

**Published:** 2026-02-14

**Authors:** Xin-Run Wang, Hao Li, Yi-Fan Hu, Ye-Xin Luo, Cheng-Fang Sun, Xin-Xin Zhang, Xin-Yi Cheng, Hua-Long Zhu, Yong-Wei Xiong, Hua Wang

**Affiliations:** 1 Department of Toxicology, Center for Big Data and Population Health of IHM, School of Public Health, Anhui Medical University, Hefei 230032, China; 2345010580@stu.ahmu.edu.cn (X.-R.W.);; 2 Key Laboratory of Environmental Toxicology of Anhui Higher Education Institutes, Hefei 230032, China; 3 Department of Preventive Medicine, School of Public Health, Wannan Medical College, Wuhu 241000, China; 4 Key Laboratory of Population Health Across Life Cycle, Anhui Medical University, Ministry of Education of the People’s Republic of China, Hefei 230032, China

**Keywords:** environmental hazards, epididymal immune microenvironment, male reproductive toxicity, male infertility

## Abstract

Environmental hazard-induced male infertility has become a major public health issue. The concealment and accumulation of environmental hazards, and their interactions with the endogenous immune network, have long been underappreciated. As the central organ for sperm maturation and motility acquisition, the epididymis plays a vital role in male fertility, and the homeostasis of the epididymal immune microenvironment (EIM) is essential. Nevertheless, a systematic synthesis of common environmental hazards and their impact on EIM, which can lead to male infertility, remains lacking. This review comprehensively summarizes the composition, functionality, and key characteristics of the EIM and underscores its critical role in preserving male reproductive health. We further evaluate and delineate the disruption of EIM homeostasis resulting from major categories of environmental exposures—including chemical, physical, biological, and behavioral hazards—and discuss their shared pathophysiological mechanisms. By integrating evidence linking environmental insults, EIM dysregulation, and male infertility, this work aims to identify pivotal molecular mechanisms from an immunological perspective. The findings provide a mechanistic foundation for the development of targeted interventions and preventive strategies against environmental hazard-induced male infertility.

## 1. Introduction

Exposure to common environmental hazards, particularly pervasive environmental pollutants, has emerged as a critical public health issue with profound implications for human health and well-being [1,2]. It is estimated that approximately 17% of couples worldwide are affected by infertility [3], with male factors contributing to roughly 30–50% of these cases [4,5,6]. Over the past four decades, a pronounced decline in sperm quality has been observed among male populations, with exposure to environmental hazards recognized as a significant contributing factor [7,8].

The epididymis plays an indispensable role in male reproduction by providing an essential pathway for sperm maturation [9,10]. Testicular sperm are immature and only acquire motility and fertilization capability during their transit through the epididymis [11]. Furthermore, the epididymis maintains a delicate balance by preventing autoimmune reactions against sperm antigens while simultaneously initiating immune defenses against pathogens [12]. Disruption of the homeostasis between the epididymal mucosa and sperm can lead to male infertility, often triggered by diverse hazards such as infections, adverse drug effects, and environmental contaminants [12,13,14]. Therefore, understanding the epididymal immune microenvironment (EIM), its impact on sperm quality and male fertility, and elucidating the mechanisms by which exogenous environmental hazards induce EIM dysregulation are essential for revealing novel toxicological foundations of male infertility.

To conduct this review, a comprehensive literature search was performed in PubMed, Scopus, and Web of Science using keywords including “heavy metals and sperm motility”, “heavy metals and blood-epididymal barrier (BEB)”, “heavy metals and sperm”, “heavy metals and asthenospermia”, “heavy metals and epididymis”, as well as queries related to other environmental hazards and reproductive toxicity. Additional searches included terms such as “epididymal macrophages”, “epididymal T cells”, “epididymal immunity”, and “epididymal inflammation”. Duplicate publications, brief reviews, editorials, and non-mammalian studies were excluded. The abstracts of the remaining articles were independently assessed by two investigators according to the following inclusion criteria: (a) publication in English; (b) epidemiological, environmental contaminant, animal, or cell culture studies; and (c) relevance to environmental pollutants, male reproductive toxicity, or epididymal immunity.

In this review, we begin by outlining the cellular composition and key characteristics of the EIM. We then systematically examine the mechanisms through which major categories of exogenous environmental hazards—chemical, physical, and biological factors—induce damage and dysregulation in the epididymis and EIM. Additionally, we summarize and discuss the associated immunological mechanisms and common pathways, including oxidative stress, inflammatory cascades, and critical signaling pathways. It is hoped that this synthesis will provide new insights and strategic directions for the prevention and treatment of male infertility.

## 2. Epididymal Immune Microenvironment

**Figure 1:**
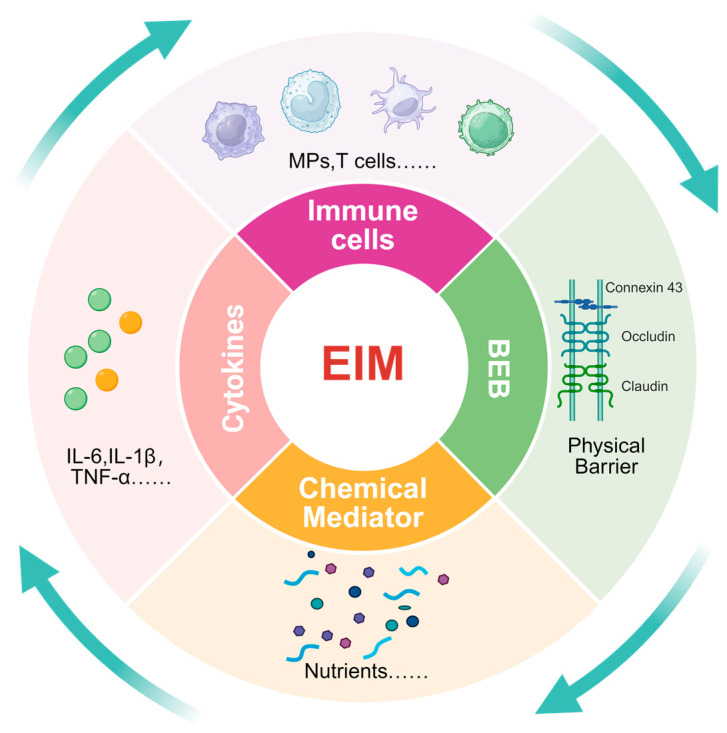
**Composition of the epididymal immune microenvironment (EIM).** The EIM comprises a complex ecosystem of immune cells, cytokines, chemical mediators, and physical barriers within and around the epididymis. This environment, through its unique immunomodulatory mechanism, ensures that sperm in the epididymis are protected from immune attack and can complete maturation. Created in BioRender. Wang, S. (2026) https://BioRender.com/h4uqlmh, accessed on 25 January 2026.

**Figure 2:**
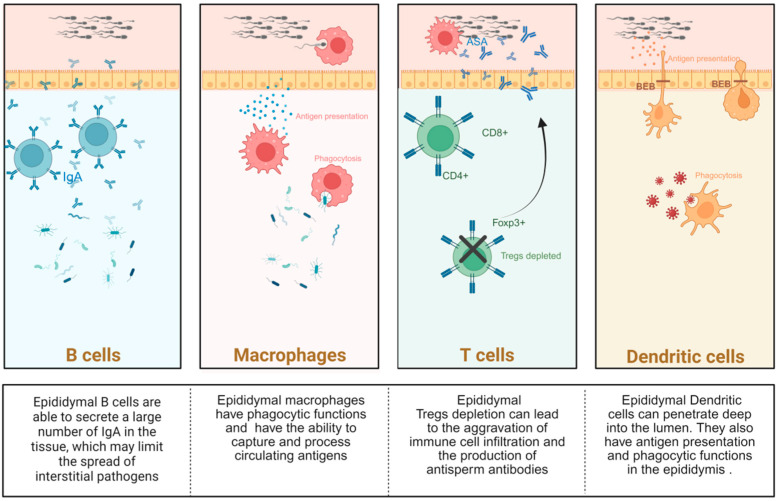
**The main immune cells in the epididymis and their characteristics.** The EIM is predominantly composed of resident and recruited immune cells, which play integral roles in maintaining local immune equilibrium and supporting spermatogenic maturation. Among these, B cells, T cells, macrophages, and dendritic cells (DCs) constitute the core cellular network that orchestrates immune surveillance, tolerance, and inflammatory regulation within the epididymis. B cells contribute to humoral immunity through antibody production, which helps neutralize pathogens while minimizing autoimmune reactivity against sperm antigens. T cells, including regulatory (Treg) and effector subsets, modulate adaptive immune responses, enforce immunologic tolerance, and participate in cytokine-mediated signaling that influences epithelial barrier function. Macrophages act as versatile sentinels, engaging in phagocytic clearance of apoptotic germ cells and microbial pathogens, and secreting immunoregulatory cytokines that shape the local inflammatory milieu. DCs serve as professional antigen-presenting cells, bridging innate and adaptive immunity by processing and presenting antigens to T cells, thereby fine-tuning immune activation versus tolerance. Created in BioRender. Wang, S. (2026) https://BioRender.com/u9vne, accessed on 25 January 2026.

The EIM is a complex and fine-grained physiological environment (Figure 1). We believe that it primarily comprises a diverse ecosystem composed of immune cells, cytokines, chemical mediators, and physical barriers within and around the epididymis (Figure 2). This environment, through its unique immunomodulatory mechanism, ensures that sperm in the epididymis are protected from immune attack and can complete the maturation process.

### 2.1. Immune Cells

#### 2.1.1. T Cells and B Cells

T cells and B cells are important members of adaptive immunity in the epididymis. A previous study has shown that the number of B cells is higher in the cauda of the epididymis than in the caput [14], and these cells can secrete a large number of local immunoglobulin A (IgA) in the tissue, which may limit the spread of interstitial pathogens [15]. Several T cell subsets, including CD4+ and CD8+, have been identified in the epididymis [16,17]. It is worth noting that Forkhead Box P3 (Foxp3)+ cells derived from CD4+ cells, namely Regulatory T cells (Tregs), have recently been shown to play a unique role in maintaining epididymal immune tolerance [17]. Tregs are specialized immune cells that regulate tissue homeostasis. In the male reproductive tract, preventing an autoimmune response against antigen-specific sperm while ensuring protection against stressors is a key determinant of fertility [17,18,19,20]. A study has shown that depletion of epididymal Treg cells can cause epididymal tubule atrophy and loss of immune tolerance in the epididymis [19], thereby exacerbating immune cell infiltration and antisperm antibody production, resulting in severe male subfertility [17]. Further exploration of the immune regulatory mechanism of epididymal T cell subsets in the epididymis may provide help for the treatment of infertility and the identification of potential targets for immunocontraception.

#### 2.1.2. Mononuclear Phagocytes (MPs)

MPs primarily comprise macrophages, DCs, and monocytes, as first proposed in 1972 [17,21]. Studies have shown that MPs cells are involved in complex processes of immune surveillance and inflammation in the reproductive system, regulating immune tolerance and activation in the reproductive system [22,23,24], and are an important part of the connection between innate and adaptive immunity [25,26]. MPs can also interact with non-immune cells in the epididymis [22,23]. They jointly regulate the balance between inflammation and immune tolerance [27].

Macrophages are the most abundant immune cell subset in the epididymis [14]. Similar to some non-immune cells, macrophages in different segments show different characteristics. CD64^+^ is considered to have a “macrophage” phenotype in the epididymis [28], and macrophages are significantly more abundant in the head of the epididymis than in the tail [14]. Like macrophages in other tissues, epididymal macrophages also have phagocytic functions. These cells can eliminate defective epithelial cells and abnormal sperm in the epididymis, as well as luminal pathogens [22,29,30,31]. In the epididymis, macrophages do not merely perform a scavenging function. A study has shown that CD64^+^ cells in the epididymis, which exhibit a “macrophage” phenotype, can capture and process circulating antigens [15]. Moreover, “macrophage type” CX3C chemokine receptor 1 (CX3CR1)^+^ macrophages with the function of capturing and presenting antigens were more abundant in the initial segment than in the other segments [15]. In addition, epididymal macrophages actively maintain BEB integrity [22]. It is worth noting that the presence of certain types of macrophages in the epididymis also makes the epididymis a target organ for viral infection because they may serve as storage vectors for viruses such as human immunodeficiency virus (HIV) [32,33], while testicular macrophages do not express some of the above targets, which also makes the epididymis more susceptible to pathogen invasion and infection than the testis [34].

The epididymis is the organ with the largest number of DCs known in the male reproductive tract so far [35]. DCs also exhibit distinct characteristics across epididymal segments; however, unlike epididymal macrophages, MPs with a “dendritic cell” phenotype are more common in the epididymal tail [24,36]. Studies have shown that epididymal DCs can capture and present antigens [24,35]. The immune tolerance of the epididymis to sperm requires transforming growth factor-β (TGF-β) signaling in DCs, and loss of TGF-β signaling leads to severe inflammatory lesions [24]. A population study has also shown that the number of DCs is significantly correlated with sperm quality and epididymal inflammation [37].

DCs can also rapidly respond to the destruction caused by unilateral efferent duct ligation (EDL) by phagocytosing apoptotic epithelial cells and their fragments, thereby maintaining the integrity of tight junctions in the epididymal epithelium [38,39]. Epididymal DCs can also recruit T helper 17 (Th17)/Th1 cells, and their production of interleukin-23 (IL-23) is considered to play a key role in the terminal differentiation and survival of Th17 cells [27,38,40]. It has also been shown that CD103^+^ accumulated in the epididymis can ameliorate lipopolysaccharide (LPS) -induced epididymal injury, and CD103^+^ DCs can also interact with epididymal MPs through Connexin 43 protein to enhance the immune response during epididymitis [23]. These findings further highlight the importance of DCs in epididymal immune regulation and maintenance of immune microenvironment homeostasis.

### 2.2. Non-Immune Cells

#### 2.2.1. Principal Cells

Principal cells, which can be directly involved in the regulation of the epididymal lumen, have a Power of Hydrogen (pH) [11]. Studies have shown that changes in epididymal lumen pH can affect sperm maturation [41,42]. In addition, it has been previously shown that the Wnt/Glycogen Synthase Kinase 3 (GSK3) signaling pathway in epididymal principal cells plays an important role in promoting sperm maturation [43]. Epididymal exosomes are mainly produced by principal cells. They are specialized cargo-delivery vesicles secreted by cells via fusion of multivesicular bodies (MVBs) with the plasma membrane (PM) [44]. Epididymal exosomes have been shown to contain hundreds of distinct protein classes, and proteins produced in different segments differ [45]. Epididymal principal cells release exosomes to transport nutrients and factors to promote epididymal sperm maturation and epithelial cell communication. Increasing evidence suggests that these exosomes may be part of the paternal epigenetic mechanism [46,47,48,49].

#### 2.2.2. Basal Cells

Epididymal basal cells exhibit segment-specific variations and, within the head region, are capable of traversing the blood–epididymis barrier (BEB) to access the luminal space [50]. These cells are recognized as an integral component of the BEB [51]. Single-cell sequencing further demonstrates that epididymal basal cells highly express genes associated with membrane transport and lipid metabolism [52]. Epididymal basal cells function as specialized luminal sensors that extend transepithelial projections to sample the luminal microenvironment directly. Specifically, they express the angiotensin II type 2 receptor (AGTR2), detect luminal angiotensin II, and subsequently signal to adjacent clear cells via the NO/cGMP pathway, thereby stimulating V-ATPase-dependent proton secretion and modulating luminal acidification, which is crucial for sperm maturation and storage [50].

#### 2.2.3. Clear Cells

A study has shown that the primary function of epididymal clear cells is to regulate the acidic pH of the epididymal lumen, thereby maintaining an appropriate luminal microenvironment before sperm maturation [53]. This function is consistent with that of principal cells in some aspects. A single-cell sequencing study has shown that genes involved in metabolic energy production are highly expressed in clear cells, which may be due to the high level of Adenosine Triphosphate (ATP) required to maintain the acidic lumenal environment [52]. The role of the interaction between epididymal principal cells and clear cells in regulating the epididymal lumen microenvironment and sperm maturation has attracted considerable attention in recent years [54,55,56].

#### 2.2.4. Narrow Cells

Epididymal narrow cells are also thought to be involved in the regulation of epididymal luminal pH [11]. A study has shown that they only exist in the IS of the epididymis [11]. At present, there are relatively few studies on narrow cells. In the future, researchers can delve deeper into the possible role of these cells in maintaining the homeostasis of the epididymis.

### 2.3. Blood–Epididymal Barrier

In the epididymis, BEB is formed by tight junctions between epididymal epithelial cells, which create a unique luminal environment for sperm maturation and protect sperm from the immune system [57,58]. Compared with the blood–testis barrier (BTB), the connection strength of BEB is far less than that of BTB [57], and the epididymis is more susceptible to pathogen invasion than the testis. BEB in different segments of the epididymis exhibited distinct characteristics. The structure of the BEB in the proximal epididymis is more complex [58,59,60]. BEB is also a highly dynamic barrier structure [61], which is the same as BTB. The function of BEB also declines with age, and tight junction protein levels in epididymal epithelial cells decrease significantly with aging [62].

The reason why BEB is called the barrier of EIM mainly lies in its protective effect on sperm and its stable state. BEB can effectively prevent sperm antigen from spilling over from the lumen and prevent immune cells from infiltrating into the lumen [14]. Under physiological conditions, immune cells are not typically detected in the epididymal lumen, and immunoglobulins are unable to cross the BEB [63,64,65]. Accompanied by immune cell infiltration and increased inflammation levels, EIM was significantly unbalanced after BEB destruction [66]. In addition, p38 mitogen-activated protein kinase (MAPK), MAPK/extracellular regulated protein kinases (ERK), TGF-β, and other signaling pathways have been shown to regulate BEB [66,67]. BEB-related gene and protein expression has also been shown to be downregulated in male azoospermia in human samples and in vitro [68,69].

### 2.4. The Uniqueness of the Epididymal Immune Microenvironment

#### 2.4.1. Regional Heterogeneity

**Figure 3:**
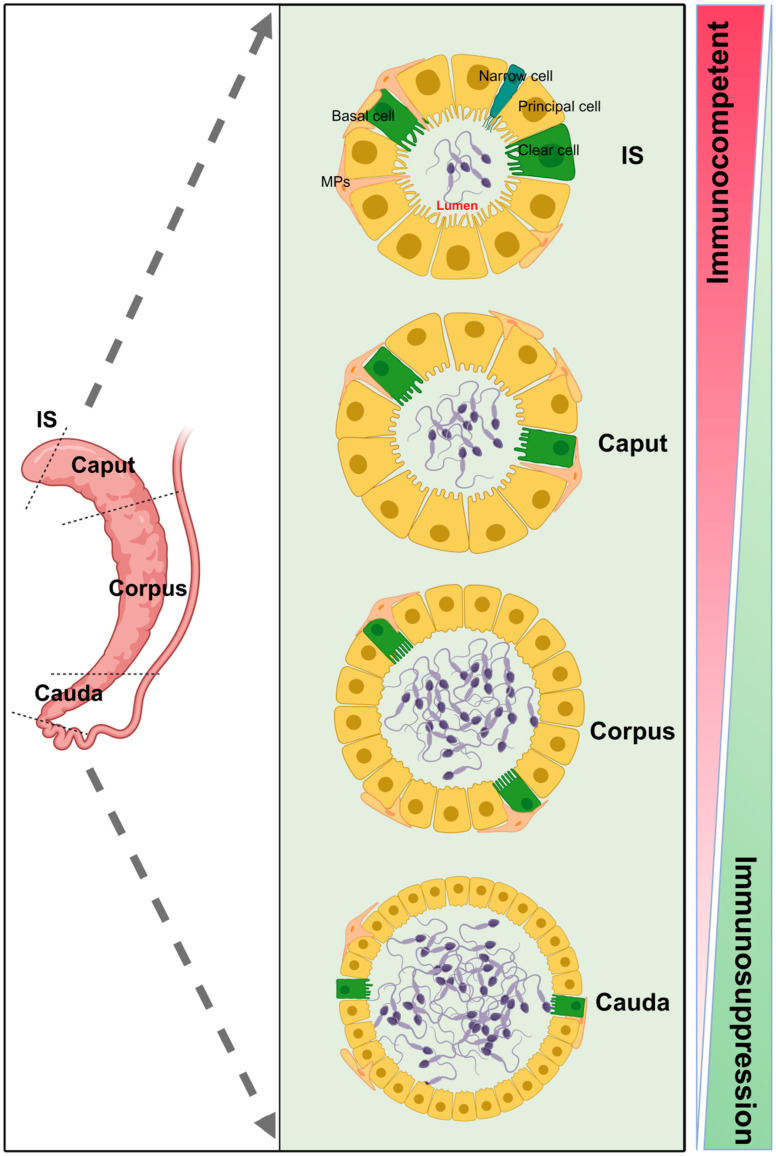
**The morphological and immune regional heterogeneity of the epididymis.** The four segments of the epididymis exhibit distinct immunological and morphological characteristics. As shown in this figure, from the IS to the cauda of the epididymis, the epididymal lumen expands, and the composition of epididymal epithelial cells differs, suggesting that the physiological functions of each segment are distinct. Created in BioRender. Wang, S. (2026) https://BioRender.com/z8pivss, accessed on 25 January 2026.

Due to regional heterogeneity in the epididymis, its immune microenvironment exhibits the greatest differences relative to other tissues, such as the testis [15,70]. This difference is due to the unique physiological structure and function of different segments of the epididymis, which leads to the significant differentiation of immune characteristics within each segment (Figure 3) [15,71].

Specifically, the proximal region of the epididymis is one of the most immunologically active regions, and its complex immune activities are mainly focused on the maintenance of homeostasis. This function not only helps to resist the invasion of pathogens into the epididymis, but also plays a necessary regulatory role in the normal physiological activities of other parts [22,71]. However, in the tail region of stored and mature sperm, immune regulatory mechanisms undergo significant changes. This region exhibits greater immunosuppressive capacity and better protects developing and maturing sperm from external interference or destruction [11,71]. This regional difference in immune characteristics was also reflected in the BEB [14], reflecting the fine regulation of immune function in each segment of the epididymis.

#### 2.4.2. Dynamic Equilibrium

15% of male infertility is caused by immune dysregulation in the reproductive system, and the dynamic equilibrium of EIM is a unique advantage [17]. This dynamic equilibrium by physical barriers, collaboration, soluble factors regulating immune cells, and the common role of regional heterogeneity adaptation mechanism [12,15,72,73], the EIM can achieve the following main goals: first, the immune mechanism of immunity to protect sperm from autoimmune reaction; second, precision defense mechanisms can efficiently remove pathogens and potential damage to protect the integrity of the reproductive system. Finally, microenvironmental homeostasis regulation provides optimal conditions for sperm maturation and storage. This kind of dynamic balance is of great significance not only for male fertility and reproductive health but also for species maintenance, and continues to play an irreplaceable role in biology.

## 3. Disorders of the Epididymal Immune Microenvironment Induced by Common Environmental Hazards

### 3.1. Chemical Hazards

#### 3.1.1. Heavy Metals

As one of the most common pollutants in the environment, heavy metals have been shown to cause significant reproductive toxicity [74,75,76].

Cadmium (Cd) is one of the most widely studied heavy metals, with reproductive toxicity receiving increasing attention in recent years. It is well known that the general population is mainly exposed to Cd through drinking water, food, and smoking [77,78]. A cross-sectional study has shown a significant negative association between cadmium and sperm quality [79]. Researchers found that Cd exposure significantly increases the levels of IL-6, IL-1β, and IL-8 in the epididymis of pigs, and induces apoptosis and inflammation of epididymal epithelial cells by activating Raf proto-oncogene serine/threonine-protein kinase (RAF1) /MAPK/ERK kinase (MEK) and nuclear factor-kappa B (NF-κB) pathways (*p* < 0.05) [80]. Cd can also disrupt EIM by damaging BEB [81]. At the pathological level, vacuolization of the porcine epididymis epithelium can also occur after Cd exposure [80]. In addition, Cd can induce a decrease in catalase activity in the rat epididymis and increase superoxide dismutase (SOD) activity [82,83].

Arsenic (As) exposure represents a global public health concern owing to its widespread contamination of groundwater and presence in certain food products [84]. A study has demonstrated that As can markedly elevate pro-inflammatory factor levels in the rat epididymis, with persistent inflammatory states correlated with increased myeloperoxidase (MPO) activity (*p* < 0.05) [85]. These findings indicate that As exposure may compromise male reproductive function by disrupting the EIM.

In addition, some studies have shown that heavy metals such as Nickel (Ni), Manganese (Mn), and chromium (Cr) can induce inflammation and oxidative stress in the epididymis, causing reproductive toxicity, including decreased sperm motility and malformation, by disrupting EIM homeostasis [86,87,88]. Interestingly, unlike the aforementioned heavy metals, the metals Selenium (Se) and Zinc (Zn) have been shown to have significant protective effects against epididymal damage [85,89,90]. Se can reduce As-induced reproductive damage by inhibiting the levels of inflammatory factors and maintaining rats’ EIM homeostasis [85]. Zn has also been shown to protect against nickel-induced EIM disorders. Zn abrogated Ni-mediated elevation in inflammatory biomarkers, including nitric oxide (NO), TNF-α, IL-1β, as well as caspase-3 activity (*p* < 0.05) [91]. In summary, it is self-evident that many heavy metals, as important reproductive poisons, can damage the epididymis. However, we should not overlook the protective role of certain metals, such as Zn, in maintaining EIM homeostasis and protecting sperm.

#### 3.1.2. Bisphenol A (BPA)

Since bisphenol pollutants from plastic use have been shown to be harmful to human health [92,93], a study has shown that BPA exposure in adolescent mice can cause male infertility by blocking epididymal immune responses [94]. After BPA exposure, basal cell projection into the lumen is reduced, suggesting that the mice’s epididymal lumen environment may change [94]. BPA decreased the levels of anti-inflammatory and pro-inflammatory cytokines IL-10, IL-6, IFN-γ, and IL-7 in the mice epididymis, increased the levels of chemoattraction-related cytokines CCL12, CCL17, CXCL16, and monocyte chemoattractant protein-1 (MCP-1), and impaired the phagocytosis of macrophages in the epididymis (*p* < 0.05 or *p* < 0.01) [94]. The above conclusions suggest that BPA exposure may disrupt EIM by downregulating intraepithelial projections and inflammation-related cytokines, thereby impairing macrophage phagocytosis and male fertility [94].

The loss of BEB integrity is an important mechanism of BPA-induced EIM disorders. A study has shown that BPA exposure can significantly reduce the expression of BEB key proteins in rats, including ZO-1, Occludin, and Claudin-1, and is accompanied by a decline in sperm quality, further suggesting damage to male reproductive function. However, melatonin treatment exerts a protective effect [95].

#### 3.1.3. Polychlorinated Biphenyls (PCBs) and Dioxins

PCBs are lipophilic chemicals that were once used as lubricants, cutting oils, and electrical insulators. They are persistent organic pollutants (POPs) [96]. cDNA microarray analysis indicated that in the epididymis of mice exposed to PCBs (Aroclor 1254), the differentially expressed genes covered multiple functional categories and biological pathways. These included GTP binding, nucleosome assembly, ribosome, and protein disulfide isomerase. Genes differentially expressed in relation to GTP binding were highly enriched. Following PCB exposure, the abundance of GTP-binding proteins associated with tight junctions declined, and the phosphorylation levels of their downstream effector proteins were affected. Experiments demonstrated that PCBs exposure enhanced the permeability of BEB and disrupted the expression of tight junction proteins such as ZO-1 and Occludin in the mouse epididymis (*p* < 0.05) [97]. In addition to disrupting the BEB, PCBs have been found to decrease the levels of α-tocopherol, ascorbic acid, and GSH in rats, and to decrease the activities of SOD, CAT, GPx, GR, and GST, while increasing the levels of ROS and LPO. Moreover, sperm motility and quantity are found to decline (*p* < 0.05) [98]. A study has also found that exposure to PCBs can inhibit the expression of androgen and estrogen receptors [99].

Dioxin is a lipophilic compound that is resistant to biodegradation and environmental degradation, enabling it to persist in the environment for extended periods. Among dioxin pollutants, 2,3,7,8-tetrachlorodibenzo-p-dioxin (TCDD) has the greatest influence [100]. Experimental evidence has indicated that TCDD exposure increases the production of reactive oxygen species such as hydrogen peroxide in the epididymis of rats, while the activities of antioxidant enzymes such as superoxide dismutase, catalase, glutathione reductase, and glutathione peroxidase are found to be decreased in epididymal sperm and the cauda (*p* < 0.05) [101].

Both PCBs and Dioxin have been shown to induce immune disorders in humans [102,103,104]. PCBs can induce apoptosis of splenocytes in mice and cause immune dysfunction due to the imbalance of Th1/Th2 cytokines [102]. PCBs have also been shown to promote macrophage/monocyte polarization to CD163+ macrophages via the Nrf2 signaling pathway, thereby accelerating atherosclerosis [103]. Experimental evidence demonstrates that most toxic actions of TCDD are mediated through the AhR [100]. Research has found that TCDD-activated AhR regulates the mutual polarization of Treg and Th17 cells through miR to inhibit inflammation induced by pertussis toxin [104]. The above experimental results suggest that PCBs and dioxins can affect the immune system. It is worth noting that the specific effects of PCBs and dioxins on epididymal immune cells remain to be further explored.

#### 3.1.4. Pesticides

Pesticides refer to chemical substances, either natural or synthetic, which are employed to get rid of pests. In agricultural production, they play a crucial role in increasing crop yields. However, notwithstanding these benefits, pesticides still bring about damaging impacts on both the environment and humans. A large number of their compounds are toxic, bioaccumulate, and are ecologically stable [105].

Organophosphorus pesticides (OPs) are widely used to protect homes and agricultural crops from insects [106]. Experiments have confirmed that organophosphorus pesticides can reduce sperm motility in rats, suggesting that OPs may affect sperm maturation in the epididymis. Other studies have also found that organophosphorus pesticides can induce epididymal phospholipidosis, and the mechanism may be related to the inhibition of epididymal secretory phospholipase A2 (sPLA2) (*p* < 0.05) [107]. Organochlorine pesticides (OCPs) have been shown in multiple studies to impair sperm motility, reduce antioxidant enzyme levels, and induce oxidative stress in the rat epididymis [108,109,110]. Research has found that, in vitamin A deficiency, the rat reproductive system becomes more susceptible to organochlorine pesticides, whereas vitamin A supplementation provides protection [111]. In addition, various insecticides, such as atrazine and cypermethrin, have been shown to damage the rat and mouse epididymis, disrupt normal epididymal sperm maturation, reduce sperm motility, and induce oxidative stress [112,113,114]. Atrazine exposure can also increase IFN-γ and estradiol (E2) levels in the mouse epididymis and decrease dihydrotestosterone (DHT) levels (*p* < 0.05). Microscopically, it can be observed that the epididymal epithelium has shed, and some shed cells can be seen in the lumen [115].

Although research on the effects of many pesticides on the epididymal immune microenvironment is relatively limited, the mechanisms by which they induce immune disorders have been extensively studied across multiple organs and reproductive tissues. Changes in the balance of cytokines and chemokines in the epididymis and testis indicate that atrazine can attenuate inflammatory signal transduction in reproductive organs [115]. Furthermore, the herbicide propanil is found to act through the AHR-NF-κB-C/EBPβ signaling axis in T cells and dendritic cells, thereby promoting intestinal inflammation [116].

#### 3.1.5. Others

In addition, other environmental endocrine-disrupting chemicals (EDCs), such as Benzo (a) pyrene, diethylhexyl phthalate (DEHP), Perfluorooctanoic acid (PFOA), and fluoride, can damage the rat and mouse epididymis and cause infertility (*p* < 0.05) [117,118,119,120]. All the aforementioned environmental hazards have been suggested to disrupt EIM and impair male reproductive function, warranting further investigation.

### 3.2. Physical Hazards

#### 3.2.1. Radiation

Exposure of organisms to radiation may induce ionization of biological macromolecules, leading to chromosome aberrations, gene mutations, or cell death [121]. A study has shown that male infertility may be related to exposure to radiation and other environmentally harmful substances [122]. For the testis, the negative effects of radiation include increased permeability of the rat’s blood–testis barrier, changes in testicular morphology, decreased semen quality, and damage to sperm DNA integrity [123]. Radiation exposure can also damage the epididymis and affect the normal maturation of sperm. Recent studies have shown that radiation exposure in mice can lead to a reduction in the number of macrophages in the epididymis (*p* < 0.05 or *p* < 0.01) [124], and it has also been found that radiofrequency radiation exposure in rats can lead to structural changes in the epididymis, with a reduction in epithelial cells, a decrease in epididymal sperm density, and an obvious epididymal edema area after exposure. Some scholars have also found that exposing rabbits to microwave radiation, which mimics mobile phone radiation, can induce apoptosis in epididymal epithelial cells at high doses [125].

These results collectively suggest that radiation may induce EIM destruction, but the specific molecular mechanism warrants further exploration. Although radiation is often regarded as a hazard, the hazardous responses at the cellular and molecular levels are usually beneficial in inducing anti-tumor immunity and alleviating inflammation [126]. Ionizing radiation, when combined with immunomodulation, can enable tumors to be targeted by the immune system [126]. The development of radioimmunology warrants re-examination of the duality of radiation in clinical practice and in the future treatment of diseases of the male reproductive system.

#### 3.2.2. High Temperature/Light Pollution

Temperature is one of the key physical hazards affecting the behavior, growth, reproduction, and survival of all animals [127]. A high-temperature environment is closely associated with heat stress, which can alter molecular and physiological levels in the mammalian reproductive system, thereby affecting its reproductive capacity [128]. A previous prospective study found that heat exposure is significantly negatively correlated with sperm parameters in the population, and that intermittent heat exposure is more harmful to sperm than continuous heat exposure (*p* < 0.05 or *p* < 0.01). The results of this study suggest that oxidative stress may be involved in this process [129]. Animal experiments showed that heat exposure significantly reduced the epididymal weight, epididymal sperm number, and motility parameters, serum testosterone level, and elevated testicular oxidative stress in guinea pigs [128].

Light pollution is also a physical hazard faced by human beings in the context of rapid urbanization. Approximately 80% of people worldwide are affected by light pollution [130]. A retrospective cohort study found that adult men living in areas with high levels of outdoor artificial light at night (ALAN) generally had worse sperm quality and lower sperm motility indicators [131]. Animal experiments have also confirmed that long-term exposure to constant light promotes poor fertilization performance. It may be achieved by reducing epididymal contractility, and slowing gamete transport through the rat epididymis is accomplished by reducing sperm lactate dehydrogenase (LDH) activity, which may be due to the moderate aging of intraductal sperm (*p* < 0.05 or *p* < 0.01) [132]. Interestingly, another study found that constant maternal light exposure reduced the epididymal weight and the number of normal epididymal sperm in male offspring rats, but significantly increased the number of epididymal epithelial cells. Continuous light exposure also led to a significant decrease in the value of glutathione reductase (GR) in the rat epididymis of the offspring [133].

The effects of heat stress and light pollution on the immune system have been documented in multiple studies [134,135,136,137]. A study has shown that the concentrations of CD4+ and CD8+ cells vary significantly across temperatures [134]. High temperatures increase the rate of cell apoptosis in mice [135]. Heat stress disrupts the differentiation cycle of T cells and their self-activation, contributing to autoimmune diseases [136]. In addition, exposure to ALAN disturbs the biological clock in the kidney [137]. It does so by elevating REV-ERBα expression, decreasing Brain and muscle arnt-like protein 1 (BMAL1) expression, and reducing the expression of the macrophage markers CD68 and the monocyte chemoattractant factor CCL2 [137]. These markers, CD68 and CCL2, are regulated by BMAL1 and REV-ERBα [137]. The immune alterations mentioned above have not been comprehensively explored and validated in the epididymal system, thus warranting further investigation.

#### 3.2.3. Nanoparticles (NPs)

In recent years, NPs have been applied across all aspects of human life, including biology, pharmacology, medicine, chemistry, physics, materials science, engineering, and industry, and human exposure to NPs is also increasing dramatically [138]. Research has shown that NPs have multi-organ toxicity, and NPs have been confirmed to be reproductive toxic [139]. The testis and epididymis are the main targets of NPs in the genital region, and a study has shown that most NPs can reach the reproductive organs or tissues of male mice through different routes, such as the testis, epididymis, and seminiferous tubules [140].

Silica NPs have been widely used in many industries due to their unique chemical properties [141,142]. As the second most widely produced nanomaterial in the world, silica NPs have also attracted considerable attention in recent years [143]. Silica NPs are also EIM disorder-inducing toxicants. Previous studies have shown that Silica NPs exposure has significant male reproductive toxicity, resulting in decreased sperm concentration and motility in the rat and mouse epididymis, and a significant increase in sperm abnormality rate [144,145]. Silica NPs exposure also induced reproductive damage by destroying BEB [66]. Studies have found that silica NPs exposure can activate the p38 MAPK signaling pathway. The expression levels of ZO-1, Claudin1, Claudin3, and Occludin proteins in the epididymis of mice were significantly down-regulated, BEB was significantly damaged, and the expression levels of TNF-α, IL-1β, and IL-6, and the rate of sperm malformation were significantly increased (*p* < 0.05) [66].

TiO_2_ NPs are widely used. A large number of experimental evidences have shown that the photocatalytic properties of TiO_2_ NPs can cause toxic effects on multiple organs in animals [146,147,148,149,150]. The International Agency for Research on Cancer (IARC) has classified TiO_2_ NPs as “possibly carcinogenic to humans” [151], and the reproductive toxicity of TiO_2_ NPs is obvious. Research has shown that it causes significant damage to the reproductive system, including the mouse epididymis [151]. Researchers used CD-1 mice to observe the reproductive toxicity of TiO_2_ NPs by long-term gavage. The results showed that epididymal sperm concentration and motility were significantly reduced, the number of abnormal sperm in the epididymal tail increased, and nanoparticle clumps appeared in the epididymal duct. With increasing exposure dose, the epididymal index was also significantly reduced [151]. Some studies have also found that TiO_2_ NPs exposure can also lead to inflammatory cell infiltration and lumen vacuolization in the epididymal lumen [152]. These results all suggest that TiO_2_ NPs exposure can disrupt EIM homeostasis in the epididymis, damage sperm, and then lead to infertility. In addition, in vitro experiments have also shown that exposure to TiO_2_ NPs can lead to a significant increase in ROS levels in human spermatozoa [153].

### 3.3. Biological Hazards

#### 3.3.1. Bacteria

**Figure 4:**
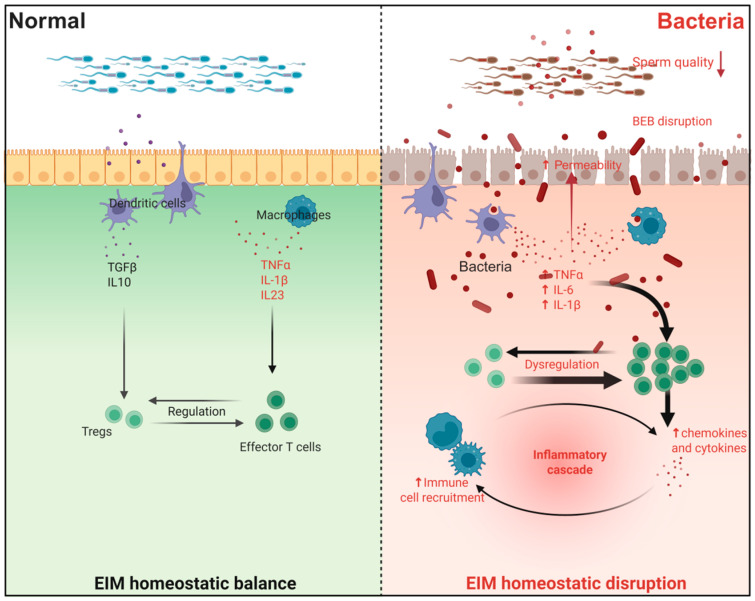
**Bacteria-induced EIM disorder.** Bacterial infection impairs the integrity of the epididymal epithelium and compromises the blood–epididymal barrier (BEB), leading to increased permeability and the translocation of immunogenic components. This breach initiates a localized immune response characterized by the infiltration and activation of immune cells, notably including pro-inflammatory T cell subsets, alongside the recruitment of monocytes/macrophages. The consequent shift in immune cell homeostasis drives a pronounced inflammatory state within the epididymal microenvironment, marked by the upregulated production and release of key inflammatory cytokines and chemokines, including TNF-α, IL-1β, IL-6, and so on. This sustained inflammatory milieu directly adversely affects sperm maturation and storage, ultimately resulting in diminished sperm motility, increased morphological abnormalities, and reduced overall sperm quality. Created in BioRender. Wang, S. (2026) https://BioRender.com/2my120a, accessed on 25 January 2026.

The human body could be exposed to harmful bacteria in the environment through a variety of ways, which can cause a variety of diseases [154]. Bacterial infection is an important cause of EIM disorder (Figure 4). In recent years, the reproductive toxicity caused by EIM disorder induced by bacterial exposure in the environment has been widely studied. Epididymitis is the most typical representative. Studies have shown that male infertility caused by epididymitis accounts for about 6% [12,34]. Clinically, the current treatment of epididymitis mainly relies on antibiotics, and it is well known that antibiotic treatment will cause a series of adverse reactions. Therefore, in-depth exploration of the specific mechanisms underlying EIM disorder induced by bacterial pathogen exposure, leading to epididymitis, is conducive to the discovery of new targets for epididymitis treatment [155]. In recent years, numerous research advances have been made in the model of induced epididymitis infection represented by *Escherichia coli* (*E. coli*) and its LPS.

*E. coli* is the most common pathogen of urinary tract infection, and more than 60% of urinary tract infections are caused by *E. coli* [156]. It has been shown that *E. coli* can significantly induce EIM disorders. At the pathological level, *E. coli* infection can initially lead to severe white blood cell infiltration in the tail of the epididymis, lumen edema, and then damage the integrity of the epididymal epithelial cells [156,157]. *E. coli* exposure can also lead to immune cell infiltration in the rat proximal epididymis [158], and cause significant increases in the levels of pro-inflammatory factors such as IL-6, TNF-α, IL-1β, etc. (*p* ≤ 0.05, *p* ≤ 0.01 or *p* ≥ 0.005), and cytokines such as Chemokine (C-X-C motif) ligand 2 (CXCL2) and C-C motif ligand 2 (CCL2) [159,160]. Notably, ROS levels in epididymal semen were significantly increased by EIM, and excessive ROS production led to a significant decrease in sperm motility [156,161], which, in turn, reduced male fertility. It has been shown that drugs such as dexamethasone can significantly alleviate an in vivo model of epididymitis caused by exposure to *E. coli* [162].

LPS exposure is the most common in vivo experimental inducer of EIM disorders. LPS can induce EIM disorder, leading to severe epididymitis, which in turn leads to male infertility. Research has shown that a variety of mechanisms may contribute to this process. Some studies have found that activation of parathyroid hormone 1 receptor (PTH1R) can alleviate LPS-induced reduction in serum testosterone level and epididymal inflammation through G Protein-Coupled Receptor-mediated q-type (Gq) and β-arrestin-1 signaling pathways, and PTH1R may be an immune regulatory target for the treatment of male reproductive system inflammation (*p* < 0.05, *p* < 0.01 or *p* < 0.001) [163]. Other studies have shown that the CX3CR1 receptor can maintain epididymal tissue homeostasis by inducing the lumen projection of epididymal MPs and regulating monocyte subsets in the epididymis in a stable state, which has a defensive effect against epididymal inflammation induced by LPS exposure [23], which is another possible immunological target for the treatment of epididymitis discovered in recent years. Dendritic cells are integral to EIM, and their normal function is essential for maintaining EIM homeostasis. Researchers found that Myd88 knockdown on dendritic cells significantly alleviated LPS-induced elevation of proinflammatory cytokines in the mouse epididymis (*p* < 0.05, *p* < 0.001, or *p* < 0.0001) [164]. Another study has found that LPS exposure can lead to a significant increase in Leptin level in the epididymis. Leptin activates the NOD-like receptor family, pyrin domain-containing 3 (NLRP3) signaling pathway to produce IL-1β production, reduces the viability of epididymal epithelial cells, and promotes cell apoptosis by activating caspase-9, caspase-3, and poly-ADP-ribose polymerase (PARP) in a concentration-dependent manner (*p* < 0.05 or *p* < 0.01). This leads to a lower fertility rate [165]. In conclusion, epididymitis induced by bacteria and their products is one of the most representative phenotypes of epididymal reproductive toxicity resulting from exposure to common environmental hazards.

#### 3.3.2. Viruses

The human body is exposed to viruses in the environment through multiple routes [166,167]. As noted above, the epididymis is susceptible to various viruses owing to its functional and structural characteristics. Studies have reported that Zika virus (ZIKV), HIV, Ebola virus (EBOV), mumps virus and other viruses have significant reproductive toxicity [168,169,170,171,172], and these viruses are one of the important exposure factors for the destruction of EIM. EIM disorders caused by epididymal virus infection are usually accompanied by immune cell infiltration, increased proinflammatory factors, BEB disruption, sperm damage, and decreased testosterone levels in mice and monkeys [169,170,172].

ZIKV can be transmitted in the population through a variety of ways, and especially through sexual transmission, which has become a public health problem. In recent years, its impact on the reproductive system has attracted wide attention. Studies have found that ZIKV can be detected in semen [173,174,175,176]. Studies have shown that ZIKV infection can alter epididymal morphology, characterized by reduced epithelial thickness and increased lumen diameter, particularly in the proximal region. Following Zika virus disruption of EIM homeostasis, inflammatory cell infiltration in the epididymis was evident, and IL-6 and IL-28 levels were significantly increased. During the month following infection with the virus, hypospermia occurs (*p* < 0.05 or *p* < 0.01) [172,173]. Notably, ZIKV can significantly reduce the expression of ZO-1 and Claudin 1 in the mouse epididymis [172], suggesting that BEB is damaged. As mentioned above, BEB contributes to the immune privilege of the epididymis and is an important part of EIM. Impairment of BEB is detrimental to the immune homeostasis required for sperm maturation.

In addition, it has been shown that the Tyrosine-protein kinase receptor (TYRO3) plays an important role in enhancing ZIKV binding to sperm [177]. In addition, for example, a small number of studies on the effects of EBOV on the reproductive system have also found that EBOV can cause necrosis of various cells in the epididymis and lead to epididymitis [171]. It is worth noting that, due to the special type of virology research, the research on the immunological mechanism of epididymal reproductive toxicity caused by various viruses is still not deep enough, and the specific treatment methods have yet to be established, which also provides opportunities for the following researchers.

### 3.4. Behavioral Hazards

Interestingly, in addition to a variety of harmful substances in the environment that can induce significant male reproductive toxicity, adverse lifestyle habits, as a component of common environmental hazards, have also been shown to cause damage to the EIM. A study has shown that long-term sleep deprivation can damage the BEB in mice and significantly downregulate the expression of tight junction proteins and androgen receptors in the epididymis. Mouse fertility decreased [178]. In recent years, the negative impact of obesity caused by a high-fat diet on male reproduction has become one of the important public health issues of people’s concern [179]. A high-fat diet has also been shown to significantly upregulate a variety of pro-inflammatory factors in the epididymis of mice, and local inflammation impairs sperm quality (*p* < 0.05, *p* < 0.01, or *p* < 0.001) [180]. High dietary fat can also lead to oxidative stress and increased apoptosis in the rat epididymis [181]. In addition, a nutrition study found that men’s consumption of ultra-processed foods can impair sperm motility in the epididymis, and this effect is not related to calorie intake [182].

## 4. Common Mechanisms of Environmental Hazards Disorder the Epididymal Immune Microenvironment

The dynamic balance of EIM depends on the integrity of the epithelial barrier, immune cell homeostasis, and the precise regulation of the oxidative-anti-inflammatory network. Although different exogenous environmental hazards have different sources and physical and chemical properties, they may break EIM homeostasis through similar molecular pathways, leading to sperm damage and male infertility. Systematically examining and elaborating these common mechanisms can not only reveal a unified treatment model across environmental hazards but also guide the development of broad-spectrum intervention strategies and inform the formulation of precise prevention and public health policies. This review summarizes the following common mechanisms, supported by detailed literature data.

### 4.1. Oxidative Stress

Reactive oxygen species (ROS) are part of normal aerobic life activities. They play a central role in reduction–oxidation (REDOX) signaling [183], and oxidative stress is associated with a variety of human diseases, including cancer, atherosclerosis, and related cardiovascular, respiratory, and neurological diseases [183]. In the reproductive system, sperm possess antioxidant defense mechanisms that neutralize ROS and protect mature sperm from oxidative damage. However, when ROS generation exceeds the antioxidant capacity of seminal plasma, oxidative stress may still occur [184]. Although moderate ROS is necessary for normal sperm function, excessive ROS production can adversely affect sperm quality and impair overall fertilization ability under pathological conditions [185]. A variety of environmental hazards have been confirmed to induce EIM disorders through oxidative stress pathways, thereby impairing sperm maturation and leading to infertility [186,187,188,189]. For example, PFOA, an emerging pollutant that has attracted considerable attention in recent years, has been shown to accumulate in the epididymis in a dose-dependent manner, resulting in decreased epididymal weight and in triglyceride (TG), cholesterol (CHO), and free fatty acid (FFA) levels. It also activated the AKT/AMPK signaling pathway in the epididymis [117]. After PFOA exposure, changes in polyunsaturated fatty acid (PUFA) composition, such as an increase in the ratio of arachidonic acid: linoleic acid (AA: LA), were observed in epididymal tissue, accompanied by excessive oxidative stress, such as an increase in malondialdehyde (MDA) and a decrease in glutathione peroxidase (GSHPx) [117]. To further explore the role of antioxidant strategies in mitigating damage to male reproductive function, many scholars have conducted antioxidant intervention studies in recent years. One study found that in rats exposed to caffeic acid (CA), Aflatoxin B1 (AFB1)-induced decrease in epididymal toxicity biomarkers (*p* < 0.05) was significantly attenuated. In addition, the decrease in antioxidant status and the increase in reactive oxygen and nitrogen (RONS) and lipid peroxidation (LPO) (*p* < 0.05) were also alleviated in CA-intervened rats [190]. CA decreased Bcl-2 expression in a dose-dependent manner. In conclusion, CA ameliorates AFB1-induced reproductive toxicity through anti-inflammatory, anti-oxidative, and anti-apoptotic mechanisms. In addition, antioxidant drugs such as anthocyanins have also been shown to improve sperm quality, including sperm count and motility, by reducing oxidative stress [191].

Although some progress has been made in related research, the spatial heterogeneity of the immune-oxidative stress regulatory network across epididymal segments and the targeting and long-term safety of existing interventions remain to be optimized. Future research can focus on advanced technologies such as spatial transcriptomics for further exploration.

### 4.2. Inflammatory Cascade

As mentioned above, the inflammatory cascade is universal and is the most common mechanism in EIM disorders induced by exposure to common environmental hazards (Table 1). Previous studies have shown that abnormal activation of the inflammatory cascade is common in the pathogenesis of EIM disorders induced by various endocrine disruptors and biological hazards [80,94,160,165,172]. This “inflammatory storm,” centered on the inflammatory signaling network and synergistically amplified by multiple pathways, is not only a common mechanism of EIM disorders caused by various environmental hazards but also a key link connecting oxidative stress and metabolic imbalance [188,192].

Toll-like receptor 4 (TLR4)/NF-κB pathway: NF-κB is a transcription factor that regulates the expression of numerous immune-related genes and is present in almost all animal cells. The TLR4/NF-κB signaling pathway, which is involved in NF-κB signaling, plays a crucial role in triggering inflammatory and immune responses, antimicrobial defense, and maintaining immune balance. The NF-κB signaling pathway is activated in response to inflammatory stimuli, including IL-1, TNF-α, and LPS. This activation process leads to an increase in the expression of key proteins such as NF-κB inhibitor protein (IκB) and p65, which in turn leads to the transfer of NF-κB to the nucleus, where it binds to DNA and promotes the release of inflammatory factors [193,194]. Dysregulation of the TLR4/NF-κB pathway induced by various environmental hazards, exemplified by LPS and EIM, has been reported to lead to male infertility in several studies [195,196,197,198]. Interestingly, the activation of this inflammatory signal can also serve as a hub that connects downstream oxidative stress and related pathways. A previous study has shown that Cd exposure can induce oxidative stress via TLR4/NF-κB signaling, thereby promoting necroptosis in the epididymis of pigs [188].

NLRP3 inflammasome: The NLRP3 inflammasome is an intracellular signaling complex that activates potent inflammatory mediators. It responds to a range of substances caused by aging, physical inactivity, overnutrition, or environmental factors, and is therefore of particular interest as a target for pharmaceutical intervention [199]. NLRP3 is a central hub in the cellular stress response because it senses a variety of stimuli that lead to loss of self-inhibition. A series of activation signals suggests that NLRP3 can sense a specific state of cellular imbalance in which its self-inhibition mechanism is relieved [199]. Several environmental hazards have been found to induce EIM disorders by activating the NLRP3 pathway [165,191,200]. Of course, there are still many potential common targets of inflammatory signaling that await further exploration and verification.

Anti-inflammatory therapy is currently the most widely used clinical intervention method for various epididymal inflammations. Developing more precise, epididymal-specific targeted interventions is a problem that future researchers need to address. For example, the significant inflammatory potential of NLRP3 and its activation in multiple scenarios of male infertility caused by environmental stress make it a highly attractive target for drug development [199].

### 4.3. Disruption of the Blood–Epididymal Barrier

The importance of BEB is that it is the first line of defense against external stressors in the epididymis, and the barrier disruption can also be considered as an injury phenotype of environmental stress. Disruption of BEB, at both the pathological and molecular levels, has been reported in nearly all studies of epididymal damage induced by environmental hazards. In this review, we argue that its disruption is an important generic hub in the induction of male reproductive impairment by environmental hazards.

The core function of BEB is to maintain the immune-exempt microenvironment in the epididymal lumen, protect sperm from immune attack, and regulate sperm maturation and storage [57,58,73]. The key pathways of BEB damage by environmental hazards are: Oxidative stress induces tight junction protein degradation, which increases the permeability of BEB [66], followed by the release of inflammatory factors, which induces further barrier damage and destroys the stability of the cytoskeleton [66,95,172]. It is worth our attention that due to the unique structure of the epididymis, the regional heterogeneity of BEB is worth further exploration. Based on current research, it is clearly insufficient. Maintaining the integrity of BEB is a key strategy for preventing male infertility induced by environmental hazards at the source. It is self-evident to understand the operation mechanism of the first line of defense, cut off this pathological chain from the source, and reduce subsequent reproductive damage. In the future, researchers should deeply understand the unique advantages of multidisciplinary collaboration and precise targeting, and explore early prevention and treatment strategies.

## 5. Conclusions and Perspectives

This review synthesizes current evidence establishing the EIM as a critical nexus where diverse environmental hazards converge to disrupt male fertility. We have detailed the sophisticated cellular architecture and regional immunological specialization of the EIM, emphasizing its essential role in sustaining sperm maturation and immunotolerance. By systematically analyzing the detrimental effects of chemical, physical, biological, and behavioral hazards, we identify oxidative stress, activation of the inflammatory cascade (notably via TLR4/NF-κB and NLRP3 pathways), and BEB dysfunction as pivotal, shared mechanistic drivers of EIM dysregulation. These converging pathways not only elucidate a unified pathological framework for environmental reproductive toxicity but also reveal actionable targets for broad-spectrum intervention strategies aimed at preserving epididymal integrity and sperm quality.

Despite these advances, the field remains predominantly descriptive, often correlating exposure with inflammatory endpoints without fully deciphering the underlying immunomodulatory networks. A significant translational gap exists between identifying these common mechanisms and developing targeted, epididymis-specific therapies. Future research must pivot toward mechanistic depth, leveraging technologies such as single-cell and spatial multi-omics to map dynamic immune cell interactions, signaling fluxes, and metabolic states within the EIM at segmental resolution. Integrating artificial intelligence with these high-dimensional datasets could predict EIM perturbation patterns and identify novel therapeutic nodes. Furthermore, there is an urgent need to develop more physiologically relevant in vitro and in vivo models that recapitulate chronic, low-dose, and multi-hazard exposures, facilitating a shift from reactive treatment to proactive, mechanism-based prevention. In addition, while this review encompasses multiple mammalian species, studies on domestic animals remain limited. Future work should also extend to livestock (e.g., cattle, sheep, goats) to better assess reproductive immunotoxicity in agriculturally and ecologically relevant species, thereby supporting veterinary health and breeding management in polluted environments.

The evaluation of emerging contaminants—including novel nanomaterials, persistent pharmaceuticals, and evolving chemical mixtures—on epididymal immunology represents both a pressing challenge and a vital opportunity. Proactive assessment of their reproductive immunotoxicity is essential for timely public health guidance. Ultimately, advancing this field requires sustained interdisciplinary collaboration across environmental toxicology, reproductive immunology, systems biology, and clinical andrology to translate mechanistic insights into effective strategies for diagnosing, preventing, and mitigating environmental infertility.

In summary, this review underscores the EIM as a fundamental determinant of male reproductive health. By framing EIM dysregulation as one of the central mechanisms in environmental infertility, we provide a conceptual foundation for innovative research and precision health strategies, aiming to safeguard male fertility in an increasingly contaminated world.

## Figures and Tables

**Table 1:** **Common mechanisms of EIM disorders caused by common environmental hazards.** Based on the author’s understanding of the current state of research on epididymal damage caused by common environmental hazards, selected published reproductive immunotoxicology studies and their main findings are listed.

Common Environmental Hazards	Animal Strain	Sample Size	Exposure Concentrations	Time and Method of Administration	Mechanisms	Male Reproductive Toxicity	Therapies
Cd [80]	Piglets	5/group	20 mg/kg	40 days: Diet	NF-κB; Inflammatory cytokines	Epididymitis; apoptosis	
As [85]	Wistar rats	10/group	60 µg AsO_2_Na L^−1^	45 days: Drinking	Inflammatory cytokines	Declined semen quality; Epididymitis	Selenium or DPDS
BPA [94]	CD-1 male mice	30/group	50 mg BPA/kg/day	6 weeks: Gavage	Macrophages dysfunction; Chemokines; Inflammatory cytokines	BEB disruption; Epididymitis; apoptosis	
PCBs [97]	Male C57 mice		5 or 500 µg/kg	50 days: Gavage	GTP	BEB disruption; Declined semen quality	
Dioxin [101]	Male Wistar rats		0.1, 1.0, or 10 µg/kg	4 days: Administered orally	Oxidative stress	Declined semen quality	
Pesticides [109]	Male Wistar rats	4/group	1, 5, and 50 mg/kg per day	45 days: Administered orally	Oxidative stress	Declined semen quality	
Perfluorooctanoic acid (PFOA) [117]	BABL/c male mice		1.25, 5, or 20 mg/kg/day	28 days: Gavage	Oxidative stress AKT/AMPK pathway	epididymis weight loss; TG, CHO, and FFA content decrease	
Radiation [124,125]	C57BL/6J		a single dose of 8.5-Gy γ-irradiation	Irradiate	Macrophages dysfunction;	Epididymitis	
	New Zealand white male rabbits	6/group	950 MHz and the output power of 3 and 6 watts	2 weeks: Irradiate	Apoptosis	Declined semen quality; BEB disruption	
High temperature [129]	Male, 22–45 years old	10/group	Testicular warming at 43 °C in a water bath, 10 times, for 30 min each time.	10 times, for 30 min each time: a water bath	Oxidative stress	Declined semen quality	
Light pollution [132]	Male Wistar rats	5/group	Constant light (L:L) for 50 days	50 days: Irradiate	sperm lactate dehydrogenase	Declined semen quality	
Silica nanoparticles [66]	C57BL/6J	15/group	0.0, 2.5, 10.0, and 20.0 mg/kg	7 days: i.p.	p38 MAPK; Inflammatory cytokines	BEB disruption; Declined semen quality	
*Escherichia coli* [156,159,165]	C57BL/6N		8000 bacteria/μL	3 days: i.p.	Th1; Inflammatory cytokines	Epididymitis	
	C57BL/6J		3 mg/kg LPS	1, 7 days: i.p.	TNF-α; Inflammatory cytokines	Epididymitis	
	Male Sprague-Dawley rats	18, 20/group	200 μg LPS	6, 12 h or 1, 2, and 3 days: i.p.	NLRP3 inflammasome; Inflammatory cytokines	Epididymitis; Declined semen quality	NLRP3 inhibitor MCC950
Viruses [170,172]	C57BL/6	4/group	10^3^ pfu ZIKV; 4 × 10^2^ pfu ZIKV	10–11 weeks, 24–30 weeks: i.p.; i.t.	Inflammatory cytokines	Epididymitis	
	AG6 mice	8/group	10^5^ pfu ZIKV	2, 5, 8 days: SC	Inflammatory cytokines	BEB disruption; Epididymitis; Declined semen quality	
High-fat diet [180]	C57BL/6	6/group	45% fat	4 weeks: Diet	Inflammatory cytokines	Epididymitis; Declined semen quality	
Ultra-processed diet [182]	Male participants	43/two group	3 weeks of an ultra-processed diet	3 weeks: Diet		Declined semen quality	Shift in dietary patterns

## Data Availability

No new data were created or analyzed in this study. Data sharing is not applicable to this article.
